# Semaphorins and Their Role in Neuropathic Pain

**DOI:** 10.3390/life16081328

**Published:** 2026-08-13

**Authors:** Mario García-Domínguez

**Affiliations:** Facultad de Educación, Universidad Alfonso X El Sabio (UAX), Avenida de la Universidad, 1 Villanueva de la Cañada, 28691 Madrid, Spain; marigado@uax.es

**Keywords:** neuropathic pain, semaphorins, neuroinflammation, sensitization, axon guidance, neuroplasticity, plexin, neuropilin

## Abstract

Neuropathic pain is a debilitating condition arising from lesions or diseases affecting the somatosensory nervous system. Despite significant advances in understanding its pathophysiology, the molecular mechanisms underlying the development and persistence of neuropathic pain remain incompletely understood. Semaphorins, a highly conserved family of axon guidance molecules, have emerged as key regulators of neuronal development, synaptic plasticity, immune responses, and neurovascular interactions. Beyond their roles during nervous system development, accumulating evidence indicates that semaphorins and their receptors, including plexins and neuropilins, actively participate in the pathological processes associated with peripheral and central sensitization following nerve injury. Altered semaphorin signaling has been implicated in neuroinflammation, aberrant axonal regeneration, glial cell activation, and dysregulated nociceptive transmission, thus contributing to the initiation and maintenance of neuropathic pain. Furthermore, several semaphorin family members show different effects, highlighting their multifaceted functions within the injured nervous system. This review summarizes the current understanding of semaphorin-mediated mechanisms involved in neuropathic pain and explores their interactions with inflammatory and neuroimmune pathways. A deeper understanding of semaphorin signaling might provide novel insights into the pathogenesis of neuropathic pain and facilitate the development of more effective treatment strategies.

## 1. Introduction

Neuropathic pain is a persistent clinical syndrome that develops following injury or dysfunction of the somatosensory system, affecting both peripheral nerves and central neural pathways [1]. Its development involves complex pathophysiological mechanisms, including peripheral and central sensitization, maladaptive neuroplasticity, dysregulated neuroimmune interactions, and maladaptive synaptic plasticity [2,3,4]. Clinically, neuropathic pain is characterized by spontaneous pain, burning or lancinating sensations, electric-shock-like episodes, allodynia, hyperalgesia, paresthesias, and, in some cases, sensory loss [5]. Importantly, these symptoms usually persist long after the initial injury, reflecting long-term maladaptive changes in neural processing rather than ongoing peripheral damage [6].

Neuropathic pain is associated with several pathological conditions, such as postherpetic neuralgia (PHN), diabetic peripheral neuropathy (DPN), stroke, multiple sclerosis, and chemotherapy-induced peripheral neuropathy (CIPN) [7,8,9,10,11,12,13]. As a result, it is a major contributor to chronic disability, psychological distress, and decreased quality of life, representing an increasing burden on healthcare systems worldwide [14,15]. Epidemiological studies estimate that neuropathic pain affects approximately 7–10% of the adult population, although prevalence varies depending on diagnostic criteria [16,17,18]. This burden is expected to continue increasing due to the rising prevalence of chronic diseases including diabetes mellitus, the leading systemic cause of neuropathic pain, as well as the growing number of cancer survivors subjected to chemotherapy and patients developing chronic pain after surgery, trauma, or herpes zoster infection [19,20,21,22,23,24,25].

At the molecular level, neuropathic pain results from maladaptive remodeling of the peripheral (PNS) and central nervous systems (CNS). In the PNS, dysregulated VGSC expression (e.g., Nav1.7 and Nav1.8), reduced K^+^ channel activity, and increased TRP channel sensitivity (e.g., TRPV1) drive primary afferent hyperexcitability [26,27,28,29]. In the CNS, long-lasting nociceptive input promotes central sensitization through enhanced glutamatergic transmission, impaired GABAergic and glycinergic inhibition, synaptic plasticity, and glial activation. Activated microglia and astrocytes release pro-inflammatory mediators, including TNF-α, IL-6, and BDNF, further sustaining neuronal hyperexcitability and chronic pain [30,31,32,33,34,35,36].

Among the molecular systems linking neuroplasticity with neuroimmune dysregulation, semaphorins have emerged as essential regulators of neuropathic pain. Originally identified as axon guidance molecules during nervous system development, semaphorins are now recognized as multifunctional proteins that regulate neuronal connectivity, axonal regeneration, synaptic organization, and glial responses in the adult nervous system [37]. Following peripheral nerve injury, dysregulated semaphorin signaling contributes to both peripheral and central sensitization by modulating neuronal excitability, synaptic remodeling, and neuroimmune communication. Acting through plexin (A/B) and neuropilin (NRP1/NRP2) receptor complexes, semaphorins activate intracellular signaling pathways involving Rho-family GTPases that govern cytoskeletal remodeling, synaptic plasticity, pro-inflammatory responses, and neuron–glia interactions, thereby enabling the establishment and maintenance of chronic pain states [38,39].

This review summarizes current evidence on the role of semaphorins in neuropathic pain, focusing on their contribution to peripheral and central sensitization after nerve injury. Also, it highlights how altered semaphorin signaling interacts with neuroinflammatory and neuroimmune pathways, driving aberrant axonal regeneration, glial activation, and dysregulated nociceptive processing. Finally, the manuscript discusses semaphorins’ potential clinical relevance and therapeutic implications.

## 2. Overview of the Semaphorin Family

### 2.1. The Semaphorin Family and Its General Functions

The semaphorin family comprises evolutionarily conserved and functionally diverse guidance cues in multicellular organisms. Initially identified as regulators of axonal guidance during embryonic development, semaphorins are now recognized as pleiotropic signaling molecules involved in a wide range of physiological processes, including nervous system connectivity, immune cell activation, vascular patterning, epithelial–mesenchymal interactions, and tissue remodeling [40,41].

Structurally, all semaphorins share a conserved extracellular sema domain, a seven-bladed β-propeller that is crucial for receptor recognition and downstream biological activity [42]. This domain is highly conserved across species and semaphorin classes, underscoring its fundamental role in semaphorin signaling [43]. In addition to the sema domain, individual semaphorin classes contain some structural features, such as immunoglobulin-like (Ig-like) domains, thrombospondin repeats, or glycosylphosphatidylinositol (GPI) anchors, which influence their localization, receptor interactions, and functional properties [44]. These structural differences determine whether semaphorins function primarily as membrane-associated, contact-dependent signals or as diffusible extracellular cues, enabling spatial and temporal regulation of cellular behavior [45]. The different components of this family, together with their functions, are shown in Table 1.

Taken together, the semaphorin family represents a complex and multifunctional signaling network that bridges developmental biology and adult tissue homeostasis. Its ability to regulate cytoskeletal dynamics, neuronal connectivity, vascular patterning, and immune responses positions it as a central regulatory system in both health and disease.

### 2.2. Semaphorin Receptors

**Figure 1:**
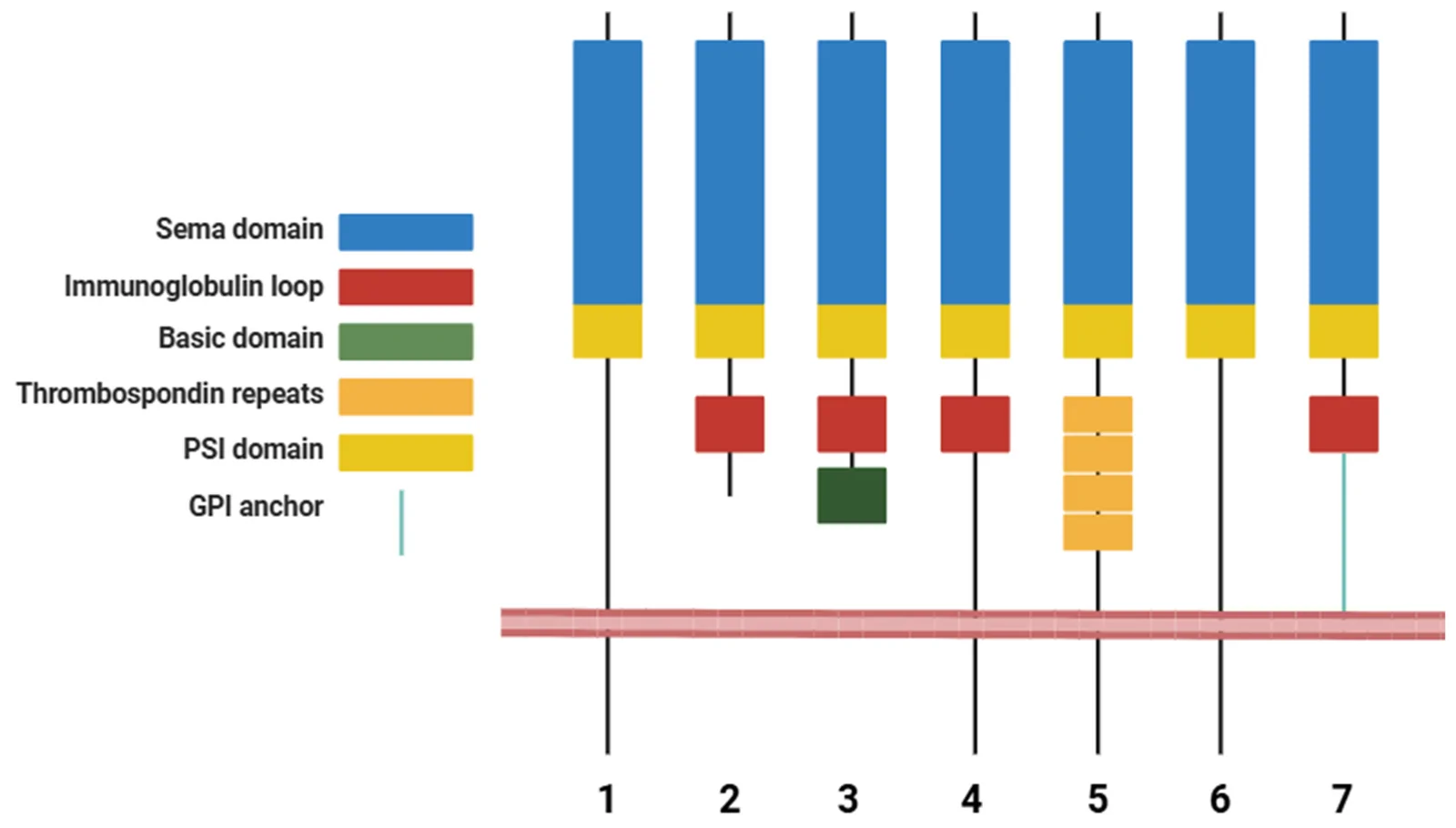
Schematic overview of the seven classes of semaphorins, showing their domain organization and the characteristic extracellular, transmembrane, and intracellular regions that underlie their diversity and signaling.

Semaphorins exert their biological functions through interactions with plexin receptors, which act as the principal signal-transducing components of the semaphorin signaling pathway [119]. In vertebrates, plexins are subdivided into four classes (A–D), each of which shows selective affinity for different semaphorin subclasses. In some cases, mainly for class 3 semaphorins, neuropilins (NRP1 and NRP2) act as co-receptors that strengthen ligand binding affinity and specificity (Figure 1 and Table 1) [120,121]. Signaling mediated by semaphorin–plexin interactions reflects a multi-scale mechanochemical transduction system in which extracellular ligand geometry is reconfigured into intracellular enzymatic switching, GTPase reprogramming, and cytoskeletal phase transitions via cooperative receptor clustering, allosteric activation of split catalytic domains, and spatial compartmentalization of signaling domains [122].

Plexin ectodomains function as ligand-dependent conformational transducers. Semaphorin binding promotes receptor dimerization and induces reorientation of the PSI-IPT domains relative to the plasma membrane [123]. Structural studies suggest that the ligand engagement restricts ectodomain flexibility, favoring plexin clustering and nanoscale receptor organization [124].

Neuropilins act as high-affinity ligand-capture co-receptors with defined binding geometry [125]. Their b1 domain interacts electrostatically with basic residues in the semaphorin C-terminus, while the a1/a2 domains contribute to plexin alignment through steric interactions [126]. NRP-plexin association relies on multiple weak interactions, generating a dynamic complex that combines rapid assembly/disassembly with high avidity and may therefore facilitate temporal filtering of transient semaphorin signals [122].

The single-pass transmembrane helix of plexin couples extracellular ligand-induced rearrangements to intracellular activation. Rather than acting as a rigid lever, it transmits conformational changes that reorganize the cytoplasmic region and relieve autoinhibition of the GAP domain [127,128]. In the inactive receptor, an autoinhibitory RBD-GAP architecture prevents formation of a functional catalytic site. Ligand-induced clustering drives trans-complementation of the split GAP domain above a critical receptor density, producing a nonlinear, switch-like response [129,130].

Plexin GAP activity promotes GTP hydrolysis through an “arginine finger” supplied by a neighboring plexin, which stabilizes the transition state by neutralizing γ-phosphate charge [131]. This leads to Rap1 inactivation, reducing RIAM/talin-dependent integrin activation and enabling disassembly of the integrin nanoclusters [132,133,134]. Simultaneously, RhoA activation induces actomyosin contractility and cortical tension [135], whereas Rac1 inhibition suppresses WAVE-Arp2/3-dependent branched actin nucleation. The resulting reduction in branched actin, together with cofilin activation and phosphoinositide depletion, favors net actin depolymerization [136,137].

CRMP proteins provide an additional cytoskeletal regulatory layer, particularly in Sema3A-neuropilin-1-plexinA signaling. CRMP2 mainly controls microtubule dynamics and directional growth, while CRMP1 enhances cytoskeletal collapse and alters actin-microtubule coordination [138,139]. Sema3A signaling activates Cdk5 and GSK3β, induces CRMP2 phosphorylation, and reduces tubulin binding, thus compromising microtubule stability and disrupting EB1/EB3-mediated +TIP tracking at the leading edge [140,141,142]. This process is coupled to RhoA-mediated actomyosin contractility, resulting in increased cortical tension [143]. CRMP1 acts as an additional amplification module that stabilizes and reinforces cytoskeletal collapse after the activation threshold is reached [144].

Overall, semaphorin signaling integrates plexin conformational activation, GAP-mediated GTPase regulation, integrin disassembly, actin remodeling, and CRMP-dependent microtubule destabilization. Collectively, these mechanisms convert extracellular semaphorin signals into coordinated cytoskeletal and mechanical outputs that regulate axon retraction, endothelial repulsion, and tissue boundary formation.

## 3. Semaphorins in the Pathogenesis of Neuropathic Pain

### 3.1. Peripheral Dysregulation of Semaphorin Signaling Following Nerve Injury

Peripheral nerve injury evokes a highly coordinated cascade of molecular events involving Wallerian degeneration, immune cell recruitment, Schwann cell dedifferentiation, axonal degeneration, and subsequent regenerative efforts [145]. While numerous growth factors and pro-inflammatory mediators (IL-1β, IL-6, and TNF-α) have been implicated in these processes, semaphorins have emerged as major regulators coordinating communication among neurons, glial cells, vascular elements, and infiltrating immune populations [39,40,41]. Their expression changes throughout the distinct phases of nerve injury, influencing whether tissue repair proceeds toward successful regeneration or advances into persistent neuropathic pain.

Under physiological conditions, semaphorins maintain neural circuit stability by restricting excessive axonal sprouting, preserving synaptic specificity, and regulating neuron–glia communication [37,38,39]. However, following injury, this regulated signaling network is disrupted because transcriptional and post-transcriptional mechanisms alter the expression of semaphorin ligands, neuropilins, plexins, and intracellular signaling mediators [146]. These molecular alterations are found not only within injured peripheral nerves but also in DRG, spinal dorsal horn, supraspinal nociceptive centers, and even peripheral immune organs, demonstrating the systemic nature of semaphorin dysregulation [147].

Among all family members, Sema3A is the most extensively studied due to its potent axonal chemorepellent activity [148]. During embryogenesis, Sema3A establishes precise guidance boundaries that suppress aberrant axonal extension [149]. Following peripheral nerve injury, however, Sema3A is upregulated in injured nerves and in multiple non-neuronal cell types, including Schwann cells, fibroblasts, and infiltrating macrophages, in the SCI and CCI preclinical models [150,151]. This injury-induced increase in Sema3A is not solely associated with axonal growth inhibition; rather, accumulating evidence indicates that Sema3A contributes to the spatial and temporal organization of regenerating axons after injury in the PNS [152,153]. On the other hand, Sema3A dose-dependently inhibits NGF-induced sprouting of CGRP- and substance P-positive fibers in the adult spinal cord, particularly at moderate NGF levels, without affecting normal sensory projections. Semaphorin3A also alleviated mechanical allodynia, supporting its potential as a therapeutic approach to limit maladaptive sensory plasticity and neuropathic pain [154].

Sema3E is increasingly recognized as a key axon guidance cue involved in the pathophysiology of nerve injury and neuropathic pain, where its expression and signaling can become dysregulated following PNS damage [155]. After nerve injury, Sema3E can be upregulated in injured tissues and associated populations, activating repulsive signaling that induces growth cone collapse, disrupts axonal regeneration, and impairs functional reinnervation of damaged pathways [156]. At the same time, Sema3E signaling interacts with the immune system, regulating macrophage activation states or NK cells that may amplify pro-nociceptive signaling in neuropathic pain [157,158].

Sema3F has emerged as an important guidance cue during peripheral nerve regeneration, contributing to the spatial organization of regenerating axons and limiting aberrant axonal sprouting [159]. Although its role in peripheral nerve injury remains less characterized than that of classical repair pathways, Sema3F signaling has been shown to modulate intracellular growth-promoting cascades, like PI3K/Akt and ERK signaling, thereby counteracting neurotrophin-mediated axonal extension [160]. This regulatory mechanism is likely to complement the injury-induced Schwann cell repair program to ensure coordinated and accurate peripheral nerve regeneration [161,162]. In this context, Sema3F signaling is thought to contribute to the balance between adaptive regeneration and aberrant sprouting that characterizes neuropathic pain states in some peripheral nerve injury models [163].

Sema4A participates in immune-neuronal communication during neuropathic pain [164]. Initially characterized as an immune semaphorin regulating T-cell activation [74], Sema4A is recognized for its participation in neuroimmune interactions within the injured nervous system [165]. Increased Sema4A expression has been linked to enhanced production of inflammatory cytokines and increased activation of macrophages [166]. Through interactions with Plexin-D1 and Tim-2 receptors, Sema4A might potentiate pro-inflammatory signaling pathways that indirectly facilitate nociceptor sensitization and long-lasting pain [167].

In the context of peripheral nerve damage, Sem5A signaling can restrict or misdirect regenerating axons, thus impairing efficient target reinnervation and contributing to incomplete functional recovery [93,95]. This regenerative constraint might indirectly favor persistent neuropathic pain by sustaining aberrant or partial reinnervation of injured territories, which is linked to nociceptor dysfunction and abnormal sensory signaling [168]. Additionally, Sema5A has been implicated in modulating local immune-stromal interactions within injured peripheral nerves, potentially shaping the inflammatory milieu that contributes to nociceptor sensitivity and axonal repair [169]. On the other hand, Sema6D can affect axonal trajectory and stabilization, thus affecting the precision of reinnervation of denervated targets [112,113]. When disrupted, this guidance activity may contribute to aberrant reinnervation, promoting nociceptor dysfunction and neuropathic pain [170].

Finally, Sema7A is upregulated in Schwann cells and infiltrating immune cells, where it operates as a bidirectional immuno-neural regulator [171]. In the context of neuropathic pain, Sema7A promotes peripheral sensitization while amplifying macrophage activation and sustaining cytokine release within the nerve tissue, all of which increase the excitability of nociceptive afferents and promote ongoing pain and hypersensitivity [172]. In parallel, Sema7A is involved in peripheral nerve regeneration, where it can exert context-dependent effects on axonal regrowth and Schwann cell behavior, influencing neurite outgrowth, guidance, and remyelination [173].

### 3.2. Involvement in CNS Neuroinflammatory Processes

**Figure 2:**
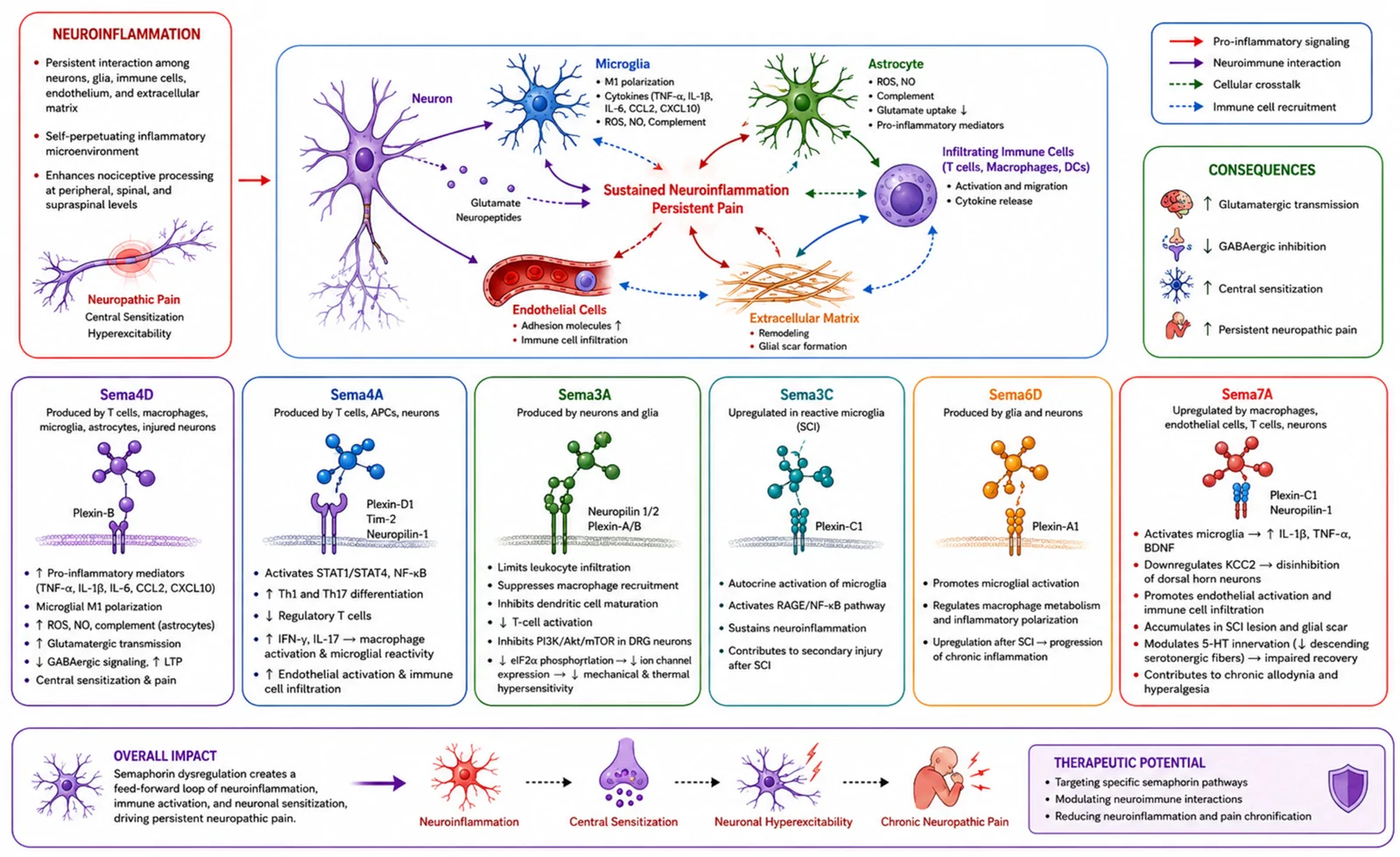
Conceptual overview of semaphorin-mediated neuroinflammatory mechanisms underlying neuropathic pain. Following peripheral nerve or CNS injury, dysregulated semaphorin signaling orchestrates bidirectional communication between neurons, microglia, astrocytes, infiltrating immune cells, endothelial cells, and extracellular matrix components, thus establishing a self-sustaining neuroinflammatory microenvironment. Various semaphorin family members regulate immune cell activation, glial polarization, cytokine production, endothelial activation, and intracellular signaling pathways, ultimately promoting neuronal hyperexcitability, impaired inhibitory neurotransmission, central sensitization, and the persistence of allodynia and hyperalgesia. Abbreviations: 5-HT (5-hydroxytryptamine -serotonin-); APCs (antigen-presenting cells); BDNF (brain-derived neurotrophic factor); CCL2 (C-C motif chemokine ligand 2); CNS (central nervous system); COX-2 (cyclooxygenase-2); CXCL10 (C-X-C motif chemokine ligand 10); DRG (dorsal root ganglion); eIF2α (eukaryotic translation initiation factor 2 alpha); GABA (γ-aminobutyric acid); IFN-γ (interferon gamma); IL-1β (interleukin 1 beta); IL-6 (interleukin 6); IL-17 (interleukin 17); iNOS (inducible nitric oxide synthase); KCC2 (potassium-chloride cotransporter 2); LTP (long-term potentiation); MAPK (mitogen-activated protein kinase); mTOR (mechanistic target of rapamycin); NF-κB (nuclear factor kappa-light-chain-enhancer of activated B cells); NO (nitric oxide); PI3K (phosphoinositide 3-kinase); RAGE (receptor for advanced glycation end products); ROS (reactive oxygen species); SCI (spinal cord injury); Sema3A (semaphorin 3A); Sema3C (semaphorin 3C); Sema4A (semaphorin 4A); Sema4D (semaphorin 4D); Sema6D (semaphorin 6D); Sema7A (semaphorin 7A); STAT (signal transducer and activator of transcription); Th (T helper cell); Tim-2 (T-cell immunoglobulin and mucin domain-containing protein 2); TNF-α (tumor necrosis factor alpha); and Treg (regulatory T cell). This image was created using ChatGPT 5.5.

Neuroinflammation has emerged as one of the key mechanisms underlying the initiation and amplification of neuropathic pain. Rather than representing a transient response to tissue injury, neuroinflammation evolves into a persistent pathological state characterized by reciprocal interactions among neurons, glial cells, infiltrating immune cells, endothelial cells, and extracellular matrix components [174,175]. This cellular network establishes a self-perpetuating inflammatory microenvironment that markedly alters nociceptive processing at peripheral, spinal, and supraspinal levels [176]. Under these conditions, semaphorins have been established as multifunctional immunoregulatory molecules that integrate neuronal and immune signaling pathways. Semaphorins are considered crucial regulators of immunity, influencing leukocyte migration, macrophage polarization, T-cell activation, dendritic cell function, cytokine secretion, angiogenesis, and glial responsiveness [147,177]. Following this, dysregulated semaphorin signaling following nerve injury contributes not only to impaired axonal regeneration but also to the establishment of a chronic inflammatory milieu, thus sustaining neuronal hyperexcitability and central sensitization (Figure 2) [178].

One of the best-characterized immunomodulatory semaphorins in neuropathic pain is Sema4D. Sema4D is distributed on T lymphocytes, microglia, astrocytes, and injured neurons [179]. CNS injury triggers Sema4D upregulation in the spinal cord, contributing to neuroimmune activation [180]. Plexin-B-mediated signaling activates various signaling pathways, resulting in increased transcription of some pro-inflammatory mediators (e.g., TNF-α, IL-1β, IL-6, CCL2, and CXCL10), iNOS, and COX-2 [181,182]. In microglial cells, Sema4D signaling promotes polarization toward the pro-inflammatory M1 phenotype, whereas astrocytic activation leads to increased production of ROS, NO, and complement factors that further amplify neuronal sensitization [182,183]. These events promote glutamatergic transmission, impair inhibitory GABAergic signaling, and facilitate LTP processes within nociceptive circuits, thereby contributing to central sensitization and persistent pain [184,185]. On the other hand, Sema4A has also emerged as an important mediator of neuroimmune communication by regulating T-cell differentiation and antigen-presenting cell function. Through interactions with Plexin-D1, Tim-2, and NRP1, Sema4A enhances activation of STAT1, STAT4, and NF-κB, favoring differentiation of Th1 and Th17 lymphocytes while suppressing regulatory T-cell activity [186]. The resulting increase in IFN-γ and IL-17 amplifies macrophage activation and microglial reactivity, processes increasingly recognized as important contributors to central sensitization following peripheral nerve injury [187]. Moreover, Sema4A promotes endothelial activation and expression of vascular adhesion molecules, facilitating immune cell infiltration into nervous tissue [188].

Sema3A exhibits a more complex and highly context-dependent role in neuroinflammation. Beyond its neuronal actions, Sema3A exerts potent immunomodulatory effects by limiting leukocyte infiltration, suppressing macrophage recruitment, inhibiting dendritic cell maturation, and reducing T-cell activation through modulation of integrin-dependent adhesion and chemokine responsiveness [189]. A recent publication reported that Sema3A limited neuropathic pain by inhibiting peripheral sensory neuron regeneration and suppressing the PI3K/Akt/mTOR signaling pathway in DRG neurons. This inhibition reduces phosphorylation of the eIF2α factor, leading to decreased ion channel expression and attenuation of mechanical and thermal hypersensitivity [190]. Sema3C has emerged as an important regulator of microglial activation following CNS injury. In the SCI murine model, Sema3C is markedly upregulated in reactive microglia, where it acts in an autocrine manner to sustain microglial activation via the RAGE/NF-κB signaling pathway. This feedback mechanism is thought to amplify the neuroinflammatory response, highlighting Sema3C as a potential contributor to secondary injury processes after SCI [191].

Recent evidence has identified Sema6D as an important regulator of macrophage metabolism and inflammatory polarization. Sema6D signaling, through Plexin-A1, promotes microglial activation under neuroinflammatory conditions [111,192,193]. Huge alterations in Sema6D expression following SCI may therefore determine whether inflammation progresses toward chronicity [194]. Sema7A has also been recognized as a key immunoregulatory semaphorin whose expression is significantly upregulated following nerve injury through activated macrophages, endothelial cells, and T cells [195]. Sema7A promotes microglial activation and the release of some pro-inflammatory mediators (e.g., IL-1β and TNF-α), and BDNF, thereby contributing to the disinhibition of dorsal horn neurons by downregulating the K^+^-Cl^−^ cotransporter KCC2 [196,197,198]. Furthermore, Sema7A is upregulated after SCI, where it accumulates at the lesion site and is expressed by neurons, endothelial cells, and later by reactive astrocytes and glial scar components. Its progressive localization to the injury core and extracellular matrix suggests a crucial role in glial scar formation and astrocyte activation, indicating that Sema7A contributes to tissue remodeling after CNS injury [199]. Sema7A modulates 5-HT innervation in the spinal cord by restricting descending projections from raphe nuclei. Its deficiency leads to increased serotonergic fiber density, particularly after SCI, without affecting receptor expression, and is associated with impaired functional recovery [200]. These mechanisms reinforce neuronal hyperexcitability and facilitate the development of allodynia and hyperalgesia [201].

## 4. Therapeutic Potential of Targeting Semaphorins

### 4.1. Preclinical Findings

The growing recognition of semaphorins as regulators of neuronal plasticity, neuroimmune interactions, and axonal regeneration has positioned these molecules as promising therapeutic targets for neuropathic pain. Unlike conventional painkillers, which primarily suppress neuronal excitability, semaphorin-targeted therapies are designed to target the molecular and cellular mechanisms responsible for the development and maintenance of neuropathic pain, including aberrant axonal sprouting, neuroinflammation, maladaptive synaptic remodeling, and glial activation. Consequently, most preclinical studies have focused on modulating semaphorin signaling to restore sensory circuit organization rather than simply reducing nociceptive transmission.

Table 2 presents the available therapeutic strategies involving semaphorins that have been used to treat neuropathic pain under various preclinical conditions.

### 4.2. Clinical Research Involving Semaphorin-Targeted Therapies

Although the role of semaphorins in neuropathic pain has been predominantly characterized in experimental and preclinical models, the emerging clinical evidence supports the potential relevance of these findings to human pathology. Table 3 describes the currently available semaphorin-based therapeutic strategies that have been evaluated for the treatment of neuropathic pain across various clinical settings. In those patients with small fiber neuropathy (SFN), a Phase II randomized clinical trial (NCT04153422) evaluated i.v. immunoglobulin (Panzyga^®^) to evaluate its efficacy in increasing intraepidermal nerve fiber density (IENFD) and reducing neuropathic pain after six months of treatment. This therapeutic strategy was based on previous evidence suggesting that IVIG may promote small nerve fiber regeneration and improve symptoms in immune-mediated SFN. In addition, Sema4D has been directly targeted using the monoclonal antibody VX15/2503 in a Phase I clinical trial (NCT01764737) among patients with multiple sclerosis [207]. Treatment with single i.v. doses ranging from 1 to 20 mg/kg was tolerated, with no dose-limiting toxicities or maximum tolerated dose identified. Moreover, VX15/2503 achieved sustained target engagement, characterized by prolonged Sema4D saturation, reduced cell-surface Sema4D expression, and an overall favorable safety profile [207].

Although these studies mainly evaluated safety, biological activity, and nerve regeneration rather than analgesic efficacy, they provide translational evidence supporting the therapeutic potential of semaphorin-related pathways in neuropathic disorders.

### 4.3. Future Research Perspectives

Despite increasing evidence implicating semaphorins in neuropathic pain, important questions regarding their mechanisms of action remain unresolved. Current studies have examined individual semaphorin family members or specific experimental models, limiting the development of an integrated understanding of semaphorin signaling throughout different neuropathic pain etiologies [154,190,202,203,204,205,206]. Future investigations combining transcriptomics, proteomics, advanced imaging, and conditional knockout models will be essential to characterize the context-dependent roles of semaphorin signaling in injured peripheral nerves, DRG, spinal cord circuits, and supraspinal pain regions [208]. The integration of scRNA-seq with spatial transcriptomics will further enable the identification of cell type-specific and spatiotemporal signaling networks across the neuroimmune axis, providing insight into the cellular heterogeneity underlying neuropathic pain [209]. Similarly, expanding human DRG atlases will facilitate the translation of experimental findings by identifying conserved and disease-associated transcriptional signatures linked to semaphorin pathways [210].

Another major challenge is to define the extensive crosstalk between semaphorin signaling and other pathways involved in pain modulation, such as cytokines, neurotrophic factors, extracellular matrix components, and ion channels. Rather than functioning independently, semaphorins likely participate in integrated molecular networks that coordinate neuroinflammation, vascular remodeling, neuronal survival, and synaptic plasticity. Elucidating these interactions will improve our understanding of the molecular mechanisms driving pain chronification and may uncover novel combinatorial therapeutic strategies.

From a translational perspective, semaphorin signaling represents an attractive target for mechanism-based therapies. Selective modulation of semaphorin-receptor interactions could simultaneously influence aberrant immune activation, maladaptive neuronal plasticity, impaired axonal regeneration, and persistent nociceptive sensitization. However, despite encouraging preclinical data, clinical evidence remains scarce, and the therapeutic potential of semaphorin-targeted interventions requires validation in adequately powered clinical studies. Additionally, the pleiotropic and context-dependent functions of semaphorins highlight the importance of developing selective therapeutic approaches that preserve beneficial signaling while targeting pathogenic pathways, instead of broadly disrupting the semaphorin signaling network.

AI-driven drug discovery may accelerate the identification of new semaphorin modulators by integrating multi-omics datasets, structural biology, and predictive modeling of ligand-receptor interactions [211]. Progress in ligand engineering, antibody-based therapeutics, and peptide mimetics also offer opportunities to improve the specificity of semaphorin-targeted interventions while minimizing off-target effects [212,213,214]. In parallel, circulating and tissue-specific semaphorin expression profiles have emerged as promising biomarkers for patient stratification, disease progression, and therapeutic response, although their clinical utility remains to be established in large prospective studies. Integrating multimodal molecular profiling with biomarker discovery is expected to facilitate the development of predictive signatures that predict which patients are most likely to benefit from semaphorin-targeted therapies, thus advancing precision medicine approaches for neuropathic pain.

## 5. Conclusions

Neuropathic pain arises from a complex interplay of neuronal, immune, and vascular alterations that ultimately contribute to maladaptive plasticity within the PNS and CNS. Although substantial progress has been made in elucidating the cellular and molecular mechanisms underlying pain chronification, reliable mechanism-based treatments remain elusive, highlighting the need to identify signaling pathways that integrate these pathological processes. In this context, semaphorins have emerged as multifaceted regulators that extend far beyond their classical developmental roles and occupy a central position at the intersection of axonal remodeling, neuroinflammation, immune cell communication, glial activation, and synaptic plasticity.

The evidence summarized in this review indicates that semaphorin signaling is dynamically regulated following nerve injury and contributes to multiple stages of neuropathic pain pathogenesis. Depending on the specific ligand–receptor pair, cellular context, and disease stage, semaphorins can produce detrimental or protective effects, highlighting the context-dependent nature and functional diversity of this protein family. Numerous semaphorins promote inflammatory responses, inhibit regenerative axonal growth, enhance glial reactivity, or facilitate maladaptive neuronal remodeling, whereas others appear to limit neuroinflammation, support tissue repair, or restore neuronal homeostasis. This functional dichotomy reflects that semaphorin signaling should not be viewed as a single pathway but rather as a highly interconnected molecular network whose biological effects are determined by the local cellular and inflammatory microenvironment.

Overall, the available evidence positions semaphorins as key molecular coordinators linking neuroimmune and vascular responses following nerve injury. Their ability to integrate developmental signaling programs with mechanisms of inflammation, regeneration, and synaptic plasticity places them among the important regulators of the initiation and progression of neuropathic pain. Clarifying the context-dependent actions of semaphorin signaling is essential for elucidating the mechanisms underlying pain chronification.

## Figures and Tables

**Table 1:** Roles of semaphorins across vertebrates and invertebrates. This table summarizes the structural features, representative members, receptor interactions, and biological roles of semaphorins across different semaphorin classes. Abbreviations: Sema1A (semaphorin 1A); N/A (not applicable); Sema1B (semaphorin 1B); Sema2A (semaphorin 2A); Sema2B (semaphorin 2B); Sema3A (semaphorin 3A); NRP1 (neuropilin 1); NRP2 (neuropilin-2); Sema3B (semaphorin 3B); Sema3C (semaphorin 3C); Sema3D (semaphorin 3D); Sema3E (semaphorin 3E); Sema3F (semaphorin 3F); Sema3G (semaphorin 3G); Sema4A (semaphorin 4A); TIM-2 (T-cell immunoglobulin and mucin domain-containing protein 2); ILT-4 (immunoglobulin-like transcript 4); Sema4B (semaphorin 4B); CLCP1 (cleft lip and palate transmembrane protein 1); PSD-95 (postsynaptic density protein 95); Sema4C (semaphorin 4C); Sema4D (semaphorin 4D); MET (mesenchymal–epithelial transition receptor); CD72 (cluster of differentiation 72); GABA (gamma-aminobutyric acid); Sema4E (semaphorin 4E); Sema4F (semaphorin 4F); CNS (central nervous system); Sema4G (semaphorin 4G); Sema5A (semaphorin 5A); Sema5B (semaphorin 5B); Sema6A (semaphorin 6A); VEGFR2 (vascular endothelial growth factor receptor 2); Sema6B (semaphorin 6B); FGFR (fibroblast growth factor receptor); PPARγ (peroxisome proliferator-activated receptor gamma); Sema6C (semaphorin 6C); Sema6D (semaphorin 6D); IRF9 (interferon regulatory factor 9); Sema7A (semaphorin 7A); GPI (glycosylphosphatidylinositol); Ig (immunoglobulin).

Class	Key Structural Domains	Representative Members	Neuropilins	Plexins	Other Receptors	Biological Roles	References
1	Sema domain Cytoplasmatic tail	Sema1A	N/A	A	N/A	Contributes to the establishment of neural circuits	[46,47]
		Sema1B	N/A	A	N/A	Participates in epidermal cell positioning during morphogenesis	[48,49]
						Involved in axon guidance	
2	Sema domain Ig-like regions	Sema2A	N/A	B	N/A	Contributes to motoneurons’ development	[46,50,51,52]
		Sema2B	N/A	B	N/A	Regulates neurotransmitter release at neuromuscular junctions	
3	Sema domain Basic C-terminal region	Sema3A	NRP1	A1-A4 D1	N/A	Participates in axonal retraction	[53,54,55,56,57,58,59]
						Inhibits immune cells’ activity	
						Involved in lymphatic vessel maturation	
		Sema3B	NRP1 NRP2	A2, A4	N/A	Involved in axon guidance	[53,54,55,60,61]
						Enhances microglial phagocytosis	
		Sema3C	NRP1 NRP2	A2, A4 B1, D1	N/A	Involved in axon guidance	[53,54,55,62,63]
						Participates in cardiovascular formation	
		Sema3D	NRP1	D1	N/A	Involved in axon guidance	[53,54,55,64,65]
						Promotes endothelial cell migration	
		Sema3E	NRP1	A1-A4	N/A	Governs vascular and neurodevelopment	[53,54,55,66,67]
						Involved in angiogenesis	
		Sema3F	NRP1	D1	N/A	Promotes growth cone collapse	[53,54,55,68,69,70]
						Involved in axon guidance	
						Promotes lymphangiogenesis	
		Sema3G	NRP1 NRP2	D1	N/A	Involved in angiogenesis	[53,54,55,71,72]
						Inhibits immune cells’ activity	
4	Sema-domain Ig-like regions	Sema4A	NRP1	B1, B2 D1	TIM-2 ILT-4	Promotes growth cone collapse	[73,74,75,76,77]
						Implicated in carcinogenesis	
						Contributes to allergic responses	
		Sema4B	NRP1 NRP2	B1, B2	CLCP1 PSD-95	Enhances microglial phagocytosis	[73,78,79,80]
		Sema4C	N/A	B2	N/A	Participates in myogenic differentiation	[73,81,82,83,84]
						Involved in axon guidance	
						Promotes macrophage recruitment	
		Sema4D	NRP1	B1, B2	MET CD72	Involved in immune system’s activation	[73,85,86,87]
						Regulator of GABAergic synapse development in the hippocampus	
		Sema4E	N/A	N/A	N/A	Required for the proper guidance of branchiomotor axons to their target tissues in zebrafish	[73,88]
		Sema4F	N/A	N/A	PSD-95	Regulator of oligodendrocytes in the CNS	[73,89,90]
		Sema4G	N/A	B2	N/A	Required for cerebellar development	[91]
5	Sema domain Thrombospondin repeats	Sema5A	N/A	A1, A3 B3	N/A	Involved in axon guidance	[92,93,94,95]
						Regulator of enteric neuron connectivity	
						Inhibitor of synaptogenesis in hippocampal dentate granule cells	
		Sema5B	N/A	A1, A3	N/A	Mediates synapse elimination in hippocampal neurons	[96,97,98]
6	Sema domain Transmembrane regions	Sema6A	N/A	A2, A4	VEGFR2	Implicated in CNS development	[99,100,101,102,103,104,105]
						Involved in angiogenesis	
		Sema6B	N/A	A2, A4	FGFR PPARγ	Implicated in carcinogenesis	[99,106,107,108]
						Involved in axon guidance	
		Sema6C	N/A	A1	N/A	Implicated in carcinogenesis	[99,109,110]
		Sema6D	N/A	A1, A4	IRF9	Involved in visual processing	[99,111,112,113]
						Involved in amygdala development	
7	Sema domain GPI anchor	Sema7A	N/A	C1	Integrin β1	Contributes to the formation of neuronal connections during development	[114,115,116,117,118]
						Inhibits immune cells’ activity	

**Table 2:** Semaphorin treatments evaluated in some preclinical models of neuropathic pain. Abbreviations: Sem3A (semaphorin 3A); CCI (chronic constriction injury); i.t. (intrathecal injection); DRG (dorsal root ganglion); PI3K (phosphoinositide 3-kinase); Akt (protein kinase B); mTOR (mechanistic target of rapamycin); eIF2α (eukaryotic initiation factor 2 alpha); IB4 (isolectin I-B4); SCI (spinal cord injury); NGF (nerve growth factor); PSNL (partial sciatic nerve ligation); LC3-II (LC3-phosphatidylethanolamine conjugate); M2 (anti-inflammatory macrophages); p65 (transcription factor p65); S100A9 (S100 calcium-binding protein A9); Sema7A (semaphorin 7A); HD (Huntington’s Disease); mAb (monoclonal antibody); NF-κB (nuclear factor kappa-light-chain-enhancer of activated B cells); PEDF (pigment epithelium-derived factor); DHA (docosahexaenoic acid); BDNF (brain-derived neurotrophic factor); NGF (nerve growth factor); PEDF-R (pigment epithelium-derived factor receptor); PLA_2_ (phospholipase A_2_); TG (trigeminal ganglia); NPY (neuropeptide Y); SPRR1A (small proline-rich protein 1A); and VIP (vasoactive intestinal peptide).

Therapeutic Target	Preclinical Model Type	Proposed Mechanisms of Action	Major Therapeutic Outcomes	References
Sem3A	SCI	Sema3A provided a chemorepulsive signal that limits NGF-induced axonal growth	Sema3A counteracted nociceptive sprouting and reduced mechanical allodynia	[154]
	CCI	Sema3A inhibited the PI3K/Akt/mTOR-eIF2α pathway and reduced ion channel expression	Increasing Sema3A expression in DRG neurons alleviated neuropathic pain by reducing mechanical and thermal hypersensitivity	[190]
		Sema3A acted through its NRP1/Plexin-A co-receptor complex in the spinal dorsal horn. Sema3A appeared to counteract pathological sensory reorganization, restoring IB4-positive unmyelinated nerve terminals in lamina II	I.t. Sema3A reduced both mechanical allodynia and thermal hyperalgesia in the CCI model	[202]
Sema3E	PSNL	Sema3E blockade reduced the accumulation of DRG macrophages, while in vitro Sema3E inhibited sensory neurite outgrowth by increasing the pro-inflammatory mediator S100A9 and decreasing the neurite growth-promoting factor Sema7A	Sema3E blockade reduced mechanical hypersensitivity in PSNL mice	[203]
Sema4D	HD model	Therapeutic blockade of Sema4D with an anti-Sema4D mAb prevented its interaction with its receptors, thereby reducing inflammatory signaling, promoting neuronal outgrowth, and enhancing oligodendrocyte maturation and myelin-related support	Treatment with mAbs against Sema4D reduced striatal, cortical, and corpus callosum atrophy and prevented testicular degeneration in the YAC128 HD mouse model. It also improved anxiety-like behavior and rescued cognitive deficits, although motor deficits were not significantly affected	[204]
	CCI	Diosgenin (Sema4D inhibitor) enhanced autophagic activity, as evidenced by increased LC3-II and Beclin-1 levels and reduced p62 expression. Enhanced autophagy inhibited microglial proliferation and NF-κB p65 nuclear translocation, while promoting M2 microglial polarization, resulting in reduced neuroinflammatory signaling	Diosgenin reduced mechanical and thermal hypersensitivity, improved sciatic nerve function, and attenuated structural damage to the sciatic nerve in CCI mice	[205]
Sema7A	Injured corneal model	PEDF activated PEDF-R PLA_2_ activity, promoting DHA release DHA enhanced docosanoid synthesis and induced the expression of BDNF, NGF, and Sema7A, which collectively promoted corneal axon growth and nerve regeneration PEDF + DHA also activated a cornea-TG signaling axis, inducing NPY, SPRR1A, and VIP expression. PEDF-R inhibition with atglistatin blocked these molecular responses	PEDF + DHA enhanced corneal nerve regeneration and axonal outgrowth following injury, potentially supporting ocular surface integrity, tear production, blink reflexes, and corneal wound healing	[206]

**Table 3:** Emerging clinical and translational evidence on semaphorin-targeted strategies. Abbreviations: Sema3A (semaphorin 3A); Sema3C (semaphorin 3C); Sema3E (semaphorin 3E); Sema4A (semaphorin 4A); SFN (small fiber neuropathy); IVIG (intravenous immunoglobulin); i.v. (intravenous injection); IENFD (intraepidermal nerve fiber density); mg/kg (milligrams per kilogram); and Sema4D (semaphorin 4D).

Semaphorin Member	Neuropathic Pain Condition	Drug Employed	Clinical Trial Phase	Key Findings	References
Sema3A Sema3C Sema3E Sema4A	SFN	Panzyga (IVIG)	Phase 2	This randomized clinical trial was designed to evaluate whether i.v. Panzyga can increase IENFD value and reduce pain in patients with SFN after 6 months of treatment The rationale is based on previous evidence suggesting that IVIG may promote small nerve fiber regeneration and improve symptoms in immune-mediated SFN	[NCT04153422]
Sema4D	Multiple sclerosis	VX15/2503 (α-Sema4D)	Phase 1	Single i.v. doses of VX15/2503 were well tolerated across all tested dose levels (1–20 mg/kg), with no dose- limiting toxicities or maximum tolerated dose identified The treatment achieved sustained target engagement, producing prolonged Sema4D saturation (≥155 days at 20 mg/kg) and reducing cellular Sema4D expression, while exhibiting dose-dependent pharmacokinetics and an acceptable safety profile	[NCT01764737] [207]

## Data Availability

No new data were created or analyzed in this study. Data sharing is not applicable to this article.

## Abbreviations

The following abbreviations are used in this manuscript:

5-HT	5-hydroxytryptamine (serotonin)
+TIP	Plus-end tracking proteins
Akt/PKB	Protein kinase B
AMPA	α-amino-3-hydroxy-5-methyl-4-isoxazolepropionic acid receptor
APC	Antigen-presenting cell
Arp2/3	Actin-related protein 2/3 complex
Aβ	A-beta fibers
BDNF	Brain-derived neurotrophic factor
CCI	Chronic constriction injury
CCL2	C-C motif chemokine ligand 2
CD72	Cluster of differentiation 72
Cdk5	Cyclin-dependent kinase 5
CIPN	Chemotherapy-induced peripheral neuropathy
Cl^−^	Chloride ion
CLCP1	Cleft lip and palate transmembrane protein 1
CNS	Central nervous system
COX-2	Cyclooxygenase 2
CPSP	Central post-stroke pain
CRMP	Collapsin response mediator protein
CRMP1	Collapsin response mediator protein 1
CRMP2	Collapsin response mediator protein 2
Cryo-EM	Cryogenic electron microscopy
CXCL10	C-X-C motif chemokine ligand 10
DHA	Docosahexaenoic acid
DN4	Douleur neuropathique 4 questionnaire
DPN	Diabetic peripheral neuropathy
DRG	Dorsal root ganglion
EB1	End-binding protein 1
EB3	End-binding protein 3
eIF2α	Eukaryotic initiation Factor 2 alpha
EM	Electron microscopy
ERK	Extracellular signal-regulated kinase
FGFR	Fibroblast growth factor receptor
GABA	Gamma-aminobutyric acid
GAP	GTPase-activating protein
GDP	Guanosine diphosphate
GPI	Glycosylphosphatidylinositol
GSK3β	Glycogen synthase kinase 3 beta
GTP	Guanosine triphosphate
GTPase	Guanosine triphosphatase
HD	Huntington’s Disease
ID-Pain	Identification of neuropathic pain questionnaire
i.t.	Intrathecal injection
i.v.	Intravenous injection
IB4	Isolectin I-B4
IENFD	Intraepidermal nerve fiber density
Ig	Immunoglobulin
IFN-γ	Interferon gamma
IL-1β	Interleukin 1 beta
IL-17	Interleukin 17
IL-6	Interleukin 6
ILT-4	Immunoglobulin-like transcript 4
iNOS	Inducible nitric oxide synthase
IRF9	Interferon regulatory factor 9
IVIG	Intravenous immunoglobulin
K^+^	Potassium ion
KCC2	Potassium-chloride cotransporter 2
LC3-II	LC3-phosphatidylethanolamine conjugate
LTP	Long-term potentiation
M1	Pro-inflammatory macrophage
M2	Anti-inflammatory macrophage
mAb	Monoclonal antibody
MAPK	Mitogen-activated protein kinase
mg/kg	Milligrams per kilogram
MET	Mesenchymal–epithelial transition receptor
mTOR	Mechanistic target of rapamycin pathway
N/A	Not applicable
Nav1.7	Voltage-gated sodium channel subtype 1.7
Nav1.8	Voltage-gated sodium channel subtype 1.8
NF-κB	Nuclear factor kappa-light-chain-enhancer of activated B cells
NGF	Nerve growth factor
NK	Natural killer cell
NMDA	N-Methyl-D-Aspartate receptor
NPY	Neuropeptide Y
NO	Nitric oxide
NRP	Neuropilin
NRP1	Neuropilin 1
NRP2	Neuropilin 2
p65	Transcription factor p65
painDETECT	PainDETECT questionnaire
PEDF	Pigment epithelium-derived factor
PEDF-R	Pigment epithelium-derived factor receptor
PHN	Postherpetic neuralgia
PI3K	Phosphoinositide 3-kinase
PLA_2_	Phospholipase A_2_
PNS	Peripheral nervous system
PPARγ	Peroxisome proliferator-activated receptor gamma
PSD-95	Postsynaptic density protein 95
PSNL	Partial sciatic nerve ligation
PSI-IPT	Plexin–semaphorin–integrin (PSI)/Immunoglobulin-like, plexin, transcription factor (IPT)
Rac1	Ras-related C3 botulinum toxin substrate 1
Rap1	Ras-related protein 1
RAGE	Receptor for advanced glycation end-products
RBD	Ras-binding domain
RhoA	Ras homolog family member A
RIAM	Rap1-GTP-interacting adaptor molecule
ROS	Reactive oxygen species
SCI	Spinal cord injury
scRNA-seq	Single-cell RNA sequencing
Sema1A	Semaphorin 1A
Sema1B	Semaphorin 1B
Sema2A	Semaphorin 2A
Sema2B	Semaphorin 2B
Sema3A	Semaphorin 3A
Sema3B	Semaphorin 3B
Sema3C	Semaphorin 3C
Sema3D	Semaphorin 3D
Sema3E	Semaphorin 3E
Sema3F	Semaphorin 3F
Sema3G	Semaphorin 3G
Sema4A	Semaphorin 4A
Sema4B	Semaphorin 4B
Sema4C	Semaphorin 4C
Sema4D	Semaphorin 4D
Sema4E	Semaphorin 4E
Sema4F	Semaphorin 4F
Sema4G	Semaphorin 4G
Sema5A	Semaphorin 5A
Sema5B	Semaphorin 5B
Sema6A	Semaphorin 6A
Sema6B	Semaphorin 6B
Sema6C	Semaphorin 6C
Sema6D	Semaphorin 6D
Sema7A	Semaphorin 7A
Src	Sarcoma
S100A9	S100 calcium-binding protein A9
SFN	Small fiber neuropathy
SPRR1A	Small pro-line-rich protein 1A
STAT	Signal transducer and activator of transcription
STAT1	Signal transducer and activator of transcription 1
STAT4	Signal transducer and activator of transcription 4
TG	Trigeminal ganglia
Th	T helper cell
Th1	T helper 1 cell
Th17	T helper 17 cell
Tim-2	T-cell immunoglobulin and mucin domain-containing protein 2
TNF-α	Tumor necrosis factor alpha
TRP	Transient receptor potential
TRPV1	Transient receptor potential vanilloid 1
VEGFR2	Vascular endothelial growth factor receptor 2
VGSC	Voltage-gated sodium channel
VIP	Vasoactive intestinal peptide
WAVE	WASP-family verprolin–homologous protein complex
